# Anesthetic Management of Direct Laryngoscopy and Dilatation of Subglottic Stenosis in a Patient with Severe Myasthenia Gravis

**DOI:** 10.1155/2012/217561

**Published:** 2012-02-02

**Authors:** Hesham A. Elsharkawy, Ursula Galway

**Affiliations:** Department of General Anesthesiology and Outcomes Research, Anesthesiology Institute, Cleveland Clinic, 9500 Euclid Avenue, Cleveland, OH 44195, USA

## Abstract

We describe the anesthetic management of a patient with severe myasthenia gravis and tracheal stenosis; the patient was scheduled for direct laryngoscopy and dilatation. The combination of myasthenia gravis and tracheal obstruction presents several difficulties for anesthetic management. The airway is shared; therefore, any complications are also shared by the anesthesiologist and bronchoscopists. The potential for respiratory compromise in patients undergoing the two procedures requires that anesthesiologists be familiar with the underlying disease state, as well as the interaction of anesthetic and nonanesthetic drugs in a case involving myasthenia gravis. We reviewed the literature and report our experience in this case. There is no strong evidence for choosing one approach to general anesthesia over another for bronchoscopy. Careful preoperative planning and experience in airway management and jet ventilation are crucial to prevent an adverse outcome and obtain favorable results.

## 1. Introduction

We present the anesthetic management of a patient with severe myasthenia gravis (MG) and tracheal stenosis she was scheduled for direct laryngoscopy and dilatation. Institutional review board (IRB) approval is not required by our institution for single case reports; therefore, written patient permission was not obtained.

 The combination of myasthenia gravis and tracheal stenosis presents several challenges for the anesthesiologist. Therefore, preoperative evaluation of the MG patient should include a review of the severity of the patient's disease and the treatment regimen. The case should therefore be reviewed with the surgeon before formulating the anesthesia plan. Specific attention should be paid to voluntary and respiratory muscle strength. The patient's ability to protect and maintain a patent airway postoperatively may be compromised if any bulbar involvement exists preoperatively. Respiratory muscle strength can be quantified by pulmonary function tests. Finally, it is critical to evaluate the severity of the subglottic stenosis and the difficulty of the intubation.

## 2. Case Presentation 

The patient was a 24-year-old female with a past medical history of myasthenia gravis (MG) and asthma. Her history included nine days of orotracheal intubation for myasthenia exacerbation. She needed five plasma phoresis exchanges and high doses of corticosteroids and azathioprine. Afterwards, the patient was discharged home in stable condition.

 At home, the MG was treated with oral pyridostigmine 60 mg, 3 times per day; prednisone 20 mg daily in the morning; oral azathioprine 75 mg twice per day. Later, she experienced about two weeks of progressive shortness of breath and stridor, which worsened with a nonproductive cough.

 She was admitted for difficulty in breathing and examined by an the ear, nose, and throat (ENT) team on arrival at the emergency department. She was found to have severe subglottic stenosis. A computerized tomography (CT) scan of the neck on admission showed that severe subglottic stenosis has developed with minimal cross-sectional diameters of 4 × 5 mm at the narrowest point (0.3^2 ^cm) approximately 75% narrowing of her trachea. Stenosis extended over a craniocaudal distance of 15 mm. Beyond the stenosis, the trachea and central airways were normal. This hour-glass configuration of the stenosis is very characteristic of endotracheal intubation injury.

 She was scheduled for microdirect laryngoscopy and tracheostomy with tracheal and subglottic dilation, injection of Depo-Medrol, and placement of mitomycin-C. The preoperative vital signs were BP 111/63; pulse 67; temperature 35.8°C (axillary); respiratory rate 20; weight 67.586 kg; SpO2 97% on room air. Airway assessment: Mallampati Score (MP) 1; neck full ROM; Airway evaluation: showed no significant abnormalities. Her voice was mildly hoarse, with mild biphasic stridor during sitting and supine position. Lung fields were clear to auscultation. Results of the neurologic and musculoskeletal examination were normal, and no bulbar weakness was evident. 

After detailed discussion with the surgeon regarding the planned surgical procedure with a patient whose recent history included MG exacerbation, we decided not use muscle relaxants. After application of the standard American Society of Anesthesiologist (ASA) monitors, we preoxygenated with 100% oxygen. General anesthesia was induced intravenously (i.v.) with midazolam 1 mg, lidocaine 60 mg, propofol 200 mg. We verified easy mask ventilation with bag and mask. General anesthesia was maintained with propofol/remifentanil infusion, and titrated with the degree of surgical stimulation and the patient's hemodynamic response. We started with propofol 300 mcg/kg/min (IV) and remifentanil 0.5 mcg/kg/min. Hydrocortisone 100 mg was given to decrease airway edema. No neuromuscular blockers were given during the surgery. 

The surgeon started with suspension laryngoscopy, sprayed lidocaine, passed the vocal cords, then completed a rigid bronchoscopy. We ventilated the lungs using jet ventilation through the ventilating bronchoscope. Initially, we started at 20 pounds per square inch (PSI). However, this was insufficient to generate a good chest rise, although oxygen saturation was in the 90s. We, therefore, decided to gradually increase the PSI to 40, with a respiratory rate of 14–18 jets per minute, allowing adequate time for exhalation. We monitored chest rise and oxygen saturation. We were able to maintain oxygen saturation between 94% and 99%. 

The patient's larynx was examined and found to be normal. Advancement of the scope to separate the true vocal folds showed good exposure of the subglottis and trachea. There was an approximately 2.5 cm stenosis about 1 cm distal to the glottis, and having 2 areas of focal webs. Her trachea was approximately 60% to 70% stenosed, which matched the preoperative CT scan. At the point, Depo-Medrol 40 mg/ML was injected into the 2 webs. Then a knife was used to make radial cuts in these 2 webs. Next, the 10–12 mm balloon was placed into the stenotic area under apnea. The patient's saturation never dropped during this time. Afterward, she was jet-ventilated without difficulty. Since we used jet ventilation during the entire procedure, we were concerned that subcutaneous emphysema would develop; but there were no clinical signs of it. 

A small mucosal tear was observed within the trachea in the posterolateral position, but it was less than 1 cm; we therefore believed it would have little effect on the patient's airway. The patient's lungs were ventilated back with bag and mask without difficulty. Surgery lasted one hour and the estimated blood loss was 20 mL. Throughout the procedure, the patient received 1 liter of crystalloid. Blood pressure was maintained with mean blood pressure (MBP) between 70 and 80 mmHg. Finally, the airway was suctioned and the patient became fully awake in the operating room. She was then transferred to the postanesthesia care unit for observation. Postoperatively, she remained in stable condition and the stridor resolved. The patient was discharged home 2 days later.

## 3. Discussion 

Myasthenia gravis (MG) is an autoimmune disease characterized by weakness and fatigability of skeletal muscles, with improvement following rest. It may be localized to specific muscle groups or generalized. It has an estimated prevalence of 1 in 20,000 [1], and affects females more than males. MG is caused by a decrease in the number of postsynaptic acetylcholine receptors at the neuromuscular junction; this decrease in turn reduces the capacity of the neuromuscular end-plate to transmit the nerve signal. Initially, in response to a stimulus resulting in depolarization, acetylcholine is released presynaptically. In MG, the number of activated postsynaptic receptors may be insufficient to trigger a muscle action potential [1]. 

 Some clinicians choose not to administer anticholinesterase on the morning of surgery, in order to minimize the need for muscle relaxants whereas others administer it for psychological support of the patient. If the patient is poorly controlled, a course of plasmapheresis may be beneficial in the preoperative period [2]. 

The steroid-dependent patient will require perioperative coverage. Anxiolytic, sedative, and opioid premedications are rarely given to patients who may have little respiratory reserve. However, if the patient has primarily ocular symptoms, a small dose of benzodiazepine is acceptable [3].

 Several general anesthetic techniques have been proposed, although none have been demonstrated as superior to the others. Some anesthesiologists prefer to avoid muscle relaxants, instead using potent inhaled agents both for facilitating tracheal intubation and providing relaxation for surgery. These agents allow neuromuscular transmission to recover, and the agents are rapidly eliminated at the end of surgery. In theory, desflurane and sevoflurane may offer some advantages, due to their low blood solubility. Sevoflurane is probably more effective than desflurane, due to its lower incidence of excitatory airway reflexes during inhalational induction [3].

 It was very challenging to keep the patient relaxed without muscle relaxants when the suspension laryngoscope was introduced and airway manipulations were stimulated. When required, small doses (10 –25% of ED 95) of intermediate-acting relaxants are titrated to the evoked MMG or EMG for both intubation and surgical relaxation. Whether or not to reverse residual neuromuscular blockade at the end of surgery is controversial.

 Response to muscle relaxants is unpredictable. Patients may be resistant to succinylcholine due to a diminished number of available receptors but sensitive to nondepolarizing agents. Therefore, muscle relaxants should be avoided, and shorter-acting drugs chosen and closely monitored. 

Some argue that anticholinesterases and antimuscarinics militate against efforts to differentiate weakness due to inadequate neuromuscular transmission from cholinergic crisis in the recovery room. They, therefore, prefer spontaneous recovery and extubation when the patient has demonstrated adequate parameters for extubation (e.g., head life, tongue protrusion) [4, 5]. Total intravenous anesthesia (TIVA) for the management of myasthenic patients has been reported. The use of remifentanil as part of TIVA may alleviate some hemodynamic instability. When feasible, many clinicians prefer to utilize regional or local anesthetic techniques [6]. Regional techniques may reduce or eliminate the need for muscle relaxant in abdominal surgery. However, jet ventilation carries its own risks, such as damage to tracheal mucosa, subcutaneous emphysema, pneumomediastinum, and pneumothorax [3].
